# Impact of the COVID-19 pandemic on early childhood development assessed with the Denver II developmental screening test in a single center

**DOI:** 10.1038/s41598-025-23761-x

**Published:** 2025-11-14

**Authors:** Selçuk Özkan

**Affiliations:** Department of Child and Adolescent Psychiatry, Kütahya Health Sciences University, Kütahya, Turkey

**Keywords:** Development, Pandemic, Children, Delay, Psychology, Health care

## Abstract

The COVID-19 pandemic has disrupted early childhood development globally by altering social environments and limiting access to early stimulation and care. While the broader consequences of the pandemic on child development have been widely speculated, objective data from structured developmental assessments remain limited. This retrospective cross-sectional study compared developmental outcomes of children aged 0 to 6 years who underwent the Denver II Developmental Screening Test at a single child psychiatry center in Kütahya, Turkey. The study included 709 children, with 431 assessed in 2019 (pre-pandemic) and 278 assessed in 2021 (during the pandemic). Developmental domains analyzed included personal-social, fine motor, language, and gross motor. Mean delay scores in the language and personal-social domains significantly increased during the pandemic period (*p* < 0.001), while fine motor delays significantly decreased (*p* < 0.001 ). No significant changes were observed in the gross motor domains or global developmental delays. Notably, the proportion of children with isolated language delays rose from 3.2% to 11.5%. Additionally, girls were found to be more affected, with significantly higher rates of developmental delays compared to boys. These findings suggest that the pandemic selectively impaired language development and personal social skills while potentially preserving or enhancing fine motor skills through increased home-based play. Public health efforts in post-pandemic recovery should prioritize early language and other interventions, especially for children and young girls exposed to the pandemic during critical language acquisition periods.

## Introduction

Early childhood is a sensitive and dynamic period during which foundational developmental domains—cognitive, motor, language, and social-emotional—undergo rapid transformation. These trajectories are shaped not only by biological maturation but also by the richness, stability, and responsiveness of the surrounding environment^1,2^. Interruptions in these domains—be they due to neglect, trauma, or environmental deprivation—can significantly alter the course of development, potentially leading to long-term delays in skill acquisition and reduced adaptive functioning throughout later childhood and adolescence.

Among the most vulnerable domains is language development, which relies heavily on reciprocal social communication and interactive linguistic input. Numerous studies have shown that both the frequency and quality of verbal interaction—particularly caregiver-child conversation and shared book reading—are crucial for expressive and receptive language growth^3,4^.

During the COVID-19 pandemic, school closures, social distancing, and increased screen time significantly reduced opportunities for such interactions, thereby posing a substantial risk to optimal language acquisition during early childhood. A scoping review of studies conducted globally since the onset of COVID-19 has shown consistent evidence of disruption in the early language environment, with particular concerns raised about delays in vocabulary growth and pragmatic language use during social isolation^3^. For example regional data from Catalonia also emphasized the cumulative impact of limited early exposure to social language contexts, showing reduced performance in expressive and receptive language among toddlers exposed to prolonged restrictions^5^.

In parallel, social interaction itself plays a fundamental role in cognitive, emotional, and behavioral regulation. Reduced peer interaction, limited emotional availability from caregivers, and disrupted daily routines can act as forms of emotional neglect, leading to elevated risk for delays in emotional, social, and language domains^6–8^. Research during the pandemic has documented increased psychological stress among caregivers, often linked to economic hardship and health concerns, which may have further compromised the emotional climate necessary for optimal child development.

Another important mechanism of developmental vulnerability during the pandemic may involve direct neurological consequences of COVID-19 exposure. Severe infection in children is rare. However, emerging neuroimaging and clinical findings suggest that even mild exposure to SARS-CoV-2 may result in subtle neurological alterations with potential cognitive and behavioral implications^9,10^.

Despite these risks, some areas of development—particularly fine motor skills—may have been relatively preserved or even improved in certain contexts. Although many studies suggest that delays in fine motor skills are common, some studies have reported the opposite, indicating potential improvements.With children spending more time at home, engaging in solitary activities like drawing, cutting, or interacting with touchscreen devices, the frequency of fine motor practice may have increased^11,12^. Evidence suggests that moderate use of tablets or structured home activities can enhance fine motor control, particularly when caregivers provide guidance and support during such tasks.

Finally, emerging data suggest that pandemic-related developmental impacts may be gender-specific. While girls typically outperform boys in early language and emotional regulation, some studies during the pandemic have reported greater declines in language performance among girls, possibly reflecting differences in stress sensitivity or social processing under conditions of isolation^13^.

This study aims to investigate the impact of the COVID-19 pandemic on early childhood development using a real-world sample of children assessed with the Denver II Developmental Screening Test in a single Turkish province. Developmental outcomes in four domains—language, personal-social, fine motor, and gross motor—were compared across two cohorts: pre-pandemic (2019) and pandemic-era (2021), with further subgroup analyses by age and sex.

## Methods

Study design and setting.

This retrospective cross-sectional study was conducted in Kütahya, a mid-sized province in western Turkey. Data were obtained from the only Child and Adolescent Psychiatry outpatient clinic in the region. The clinic serves a relatively stable population with low internal migration and birth rates consistent with national averages. The study included children aged 0–6 years who were evaluated using the Denver II Developmental Screening Test in two distinct periods: pre-pandemic (2019) and during the pandemic (2021). Ethical approval was obtained from the Ethics Committee of Kütahya Health Sciences University (Decision No: 2025/03–22; Date: February 27, 2025). Due to the retrospective nature of the study, the Ethics Committee of Kütahya Health Sciences University waived the need of obtaining informed consent.

Participants.

A total of 709 children were included in the study. “All eligible cases from these periods were included to ensure representativeness of the clinical population and adequate sample size for statistical analyses. Only children who underwent Denver II testing during their clinical evaluation were included; repeated assessments of the same child were excluded. Children were referred for testing for a variety of developmental concerns; however, diagnostic categories, specific complaints, and reasons for referral were not analyzed further in this analysis to preserve the representativeness of the sample. Children with missing data, repeated Denver II assessments, or incomplete evaluations due to non-compliance were excluded from the analysis. These exclusions were minimal and did not materially affect the final sample composition. Because the study was retrospective and the analytic process was straightforward, a formal flow diagram was not prepared. The final analytic sample therefore consisted of 431 children in 2019 and 278 children in 2021.

Measures.

The Denver II Developmental Screening Test is a standardized developmental assessment tool that evaluates children across four domains: Language, Fine Motor-Adaptive, Personal-Social, and Gross Motor. In its standard use, the test classifies children’s performance categorically (normal, questionable, or abnormal) based on established scoring rules, including “delay” (failure of an item passed by 90% of age-matched peers) and “caution” (failure of an item passed by 75–90% of peers). In our clinic, the Denver II was administered by trained child development specialists who had received formal instruction in its standardized application. The test was conducted individually with the child in a play-based setting, and additional information was obtained from parents when required. Each assessment typically lasted 20–30 min. For the purposes of this study, rather than using only the categorical classifications, we treated each failed item as an indicator of developmental delay and calculated a cumulative delay score, with higher scores reflecting a greater number of developmental delays. The test was administered by a total of three child development specialists across the two study periods, with one examiner change between 2019 and 2021. All examiners had received formal training in the Denver II and followed standardized administration protocols to ensure consistency across assessments. Although formal inter-rater reliability testing was not performed, the use of standardized training and protocols minimized variability between examiners.

### Statistical analysis

Sociodemographic variables (age in months and sex) and domain-specific Denver II scores were collected from clinical records. Children were grouped by year of assessment (2019 vs. 2021). The 2019 and 2021 cohorts consisted of independent samples; no child was included at both time points. Normality of the data was assessed using the Kolmogorov–Smirnov test. Independent samples t-tests were used to compare mean scores and additional subgroup analyses were conducted by sex. Categorical variables (e.g., gender distribution, isolated language delay, global developmental delay) were compared using chi-square tests.All analyses were conducted using SPSS version 26. Statistical significance was defined as *p* < 0.05 for all comparisons.

## Results

This study included 709 children aged 0–6 years who underwent developmental assessment using the Denver II Developmental Screening Test. Participants were divided into two groups based on the year of assessment: pre-pandemic (2019, *n* = 431) and pandemic period (2021, *n* = 278).

Table 1 presents the demographic characteristics of children who underwent Denver II developmental screening in 2019 and 2021. For each year, the total number of children, average age in months, and gender distribution are shown. There was no statistically significant difference in gender distribution between the years (*χ*²^1^ = 0.89, *p* = 0.345; OR = 1.19, 95% CI 0.85–1.66). Mean age differed by approximately 2.36 months (2019: 37.90 ± 21.71 vs. 2021: 40.25 ± 16.93), which was not statistically significant (*t*(682) = 1.62, *p* = 0.106; Cohen’s d = 0.12; mean difference = 2.36 months, 95% CI − 0.51 to 5.22).

**Table 1:** Demographic characteristics of the Sample.

Year	Total Children	Average Age	Male	Female	% Male	% Female
2019	431	37.90	296	135	68.68	31.32
2021	278	40.25	201	77	72.30	27.70

Table 2 presents the average delay scores in each of the four developmental domains assessed with the Denver II Developmental Screening Test in 2019 and 2021. Higher scores indicate greater developmental delay, while a score of 0 represents age-appropriate (normal) development. Independent sample t-tests were used to compare the scores between years.

**Table 2:** Mean delay scores across the four developmental Domains.

Developmental domain	Mean score (2019)	mean score (2021)	*p*-value
Personal-Social	1.66	2.24	0.004
Fine Motor	4.20	2.41	< 0.0001
Language	6.90	9.73	< 0.0001
Gross Motor	2.66	2.84	0.632

There was a statistically significant difference between 2019 and 2021 in three of the four developmental domains. Language delays increased (2019 M = 6.90 vs. 2021 M = 9.73; *t* = − 4.57, *p* < 0.0001; Cohen’s *d* = 0.36, 95% CI [1.61–4.05]). Personal-social delays also increased (M = 1.66 vs. 2.24; *t* = − 2.88, *p* = 0.0041; *d* = 0.24, 95% CI [0.19–0.98]), whereas fine motor delays decreased significantly (M = 4.20 vs. 2.41; *t* = 4.86, *p* < 0.0001; *d* = 0.33, 95% CI [− 2.51 to − 1.06]). Gross motor development did not show a significant change (M = 2.66 vs. 2.84; *t* = − 0.48, *p* = 0.632; *d* = 0.04, 95% CI [− 0.55–0.91]).

**Fig. 1:**
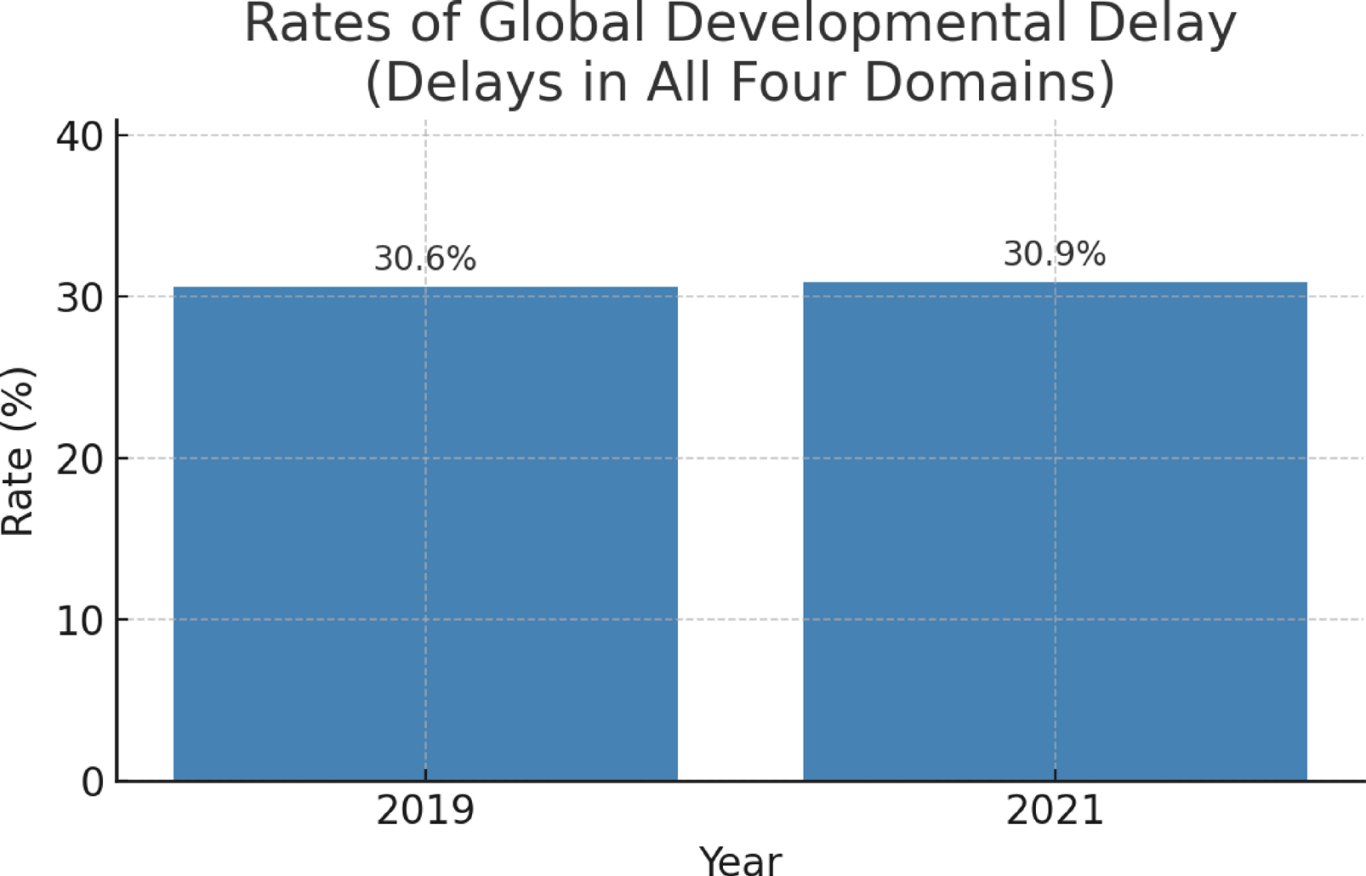
Comparison of global developmental delay rates before and during the pandemic period.

As shown in Fig. 1, the analysis conducted to assess the comparability of the overall profile of applicants across time periods, the proportion of children with global developmental delay—defined as having delays in all four developmental domains—was found to be 30.6% in the pre-pandemic period and 30.9% during the pandemic. This difference was not statistically significant (*p* = 0.997; OR = 1.01, 95% CI [0.70–1.44] ), suggesting that the overall developmental risk profile of children presenting for evaluation remained stable across periods.

**Fig. 2:**
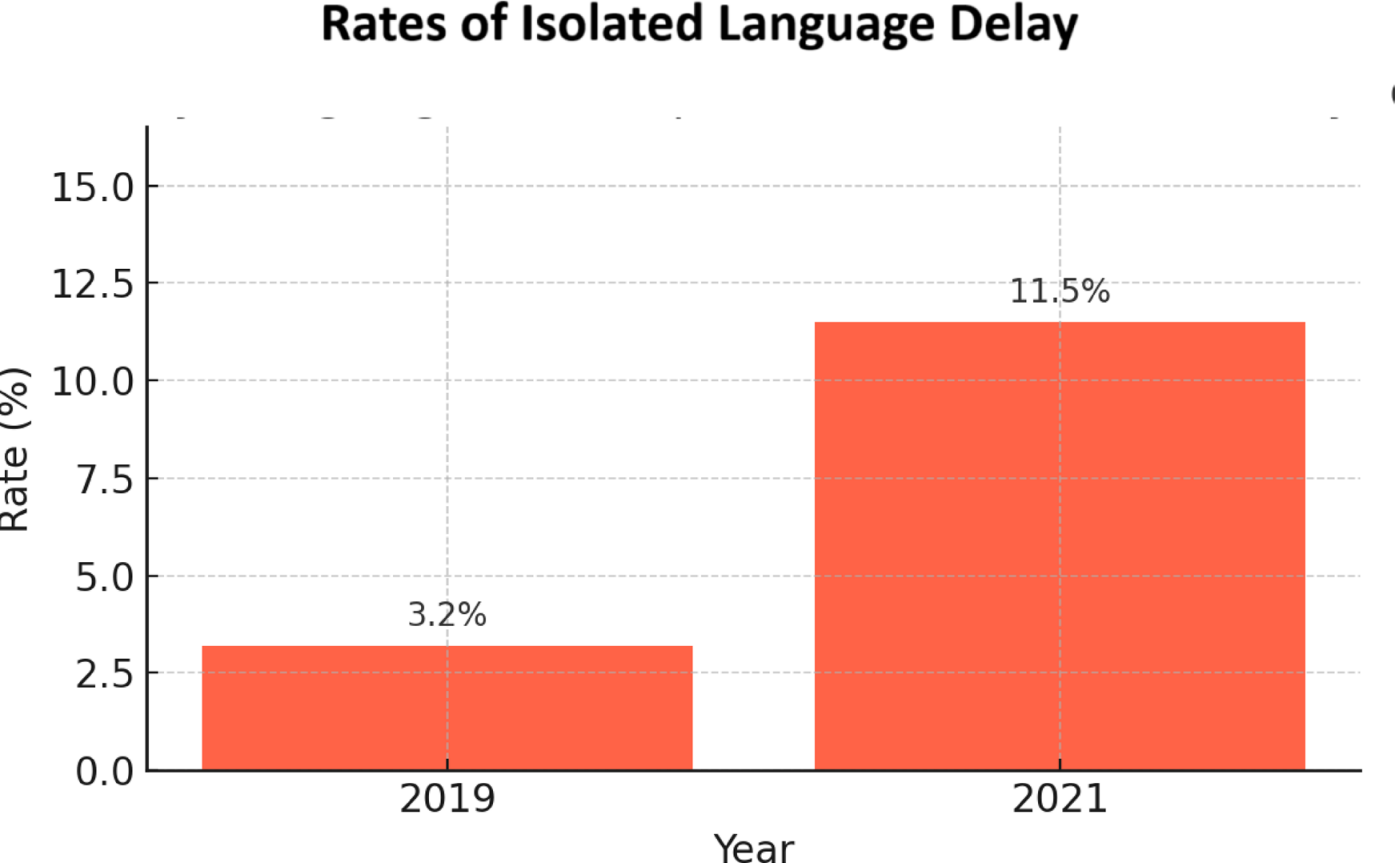
Rates of isolated language delays before and during the pandemic period.

Figure 2 presents the percentage of children showing no delay in other developmental domains (personal-social, fine motor, gross motor) but a delay in the language domain, indicating isolated language development delay. The rate of isolated language development delay significantly increased after the pandemic.

In 2019, the rate was 3.2% (14 out of 431 children), while in 2021, it rose to 11.5% (32 out of 278 children). This difference was statistically significant ( *p < 0.0001; OR = 3.87*,* 95% CI [2.03–7.40] ).*

In 2019, boys had significantly higher language delay scores compared to girls (*M* = 7.67 vs. *M* = 5.23; *p* = 0.0024 ; Cohen’s *d* = 0.32, 95% CI [0.87–4.00] ), indicating greater vulnerability in this domain. No statistically significant gender differences were observed in the personal-social, fine motor, or gross motor domains in that year (*p* > 0.05 for all).

In contrast, in 2021, girls exhibited significantly higher scores than boys in the personal-social domain (*M* = 3.00 vs. *M* = 1.96; *p* = 0.0172; Cohen’s *d* = 0.31, 95% CI [0.19–1.90] ), fine motor domain (*M* = 3.43 vs. *M* = 2.02; *p* = 0.0109; *d* = 0.34, 95% CI [0.33–2.49] ), and gross motor domain (*M* = 4.13 vs. *M* = 2.35; *p* = 0.0188; *d* = 0.36, 95% CI [0.31–3.25] ), suggesting that girls were more adversely affected in these areas during the pandemic period. Although the mean language delay score was higher in girls than boys in 2021 (*M* = 11.29 vs. *M* = 9.14), the difference did not reach statistical significance (*p* = 0.0775; *d* = 0.24, 95% CI [− 0.24–4.53] ).

These findings suggest a shift in gender vulnerability following the onset of the COVID-19 pandemic, with girls exhibiting increased delays in several developmental domains, whereas in the pre-pandemic period, boys were more affected, particularly in language development.

## Discussion

This retrospective study provides empirical evidence on the developmental impacts of the COVID-19 pandemic in early childhood. The findings reveal a clear increase in language and personal-social skills delay during the pandemic period,. These results are consistent with prior reports emphasizing developmental vulnerabilities due to reduced social interaction, disrupted routines, and heightened caregiver stress^3,5^. They underscore the importance of consistent verbal engagement and structured daily experiences in supporting early communication milestones.

A growing body of research has emphasized that the developmental impact of the COVID-19 pandemic extends beyond behavioral issues, affecting caregiving practices, environmental stability, and parent-child relational dynamics^8,14^. Social restriction measures such as lockdowns and school closures have been shown to elevate stress levels in both parents and children, increasing the risk of adverse childhood experiences, which are strongly associated with developmental delays and long-term health outcomes. Emotional unavailability during this period has been described as an “invisible form of neglect. This may contribute to language delays not only via reduced verbal exposure but also through disrupted relational connections. In line with these findings, our study revealed that children who experienced the pandemic during sensitive developmental windows—particularly in language and personal-social domains—exhibited measurable delays in developmental test scores.

A recent cohort study from Japan found that children exposed to the COVID-19 pandemic exhibited a developmental delay of approximately 4.4 months by age 5, with no significant delay observed at age 3. The study also highlighted greater developmental variability and underscored the moderating effects of nursery care quality and parental mental health^15^. Similarly, in a longitudinal study they reported persistent deficits in language development among children born and raised during the pandemic, including impairments in word segmentation, learning, and lexical growth between 12 and 24 months^16^. These findings align with our results, where the language domain was the most negatively affected, reinforcing the notion that early language acquisition is particularly sensitive to disruptions in environmental stimulation and social interaction.

Importantly, our results did not show substantial deterioration in gross motor domain, suggesting that not all developmental areas were equally affected. In many Turkish households, multigenerational living and parental proximity may have buffered some of the social impacts experienced elsewhere. However, this may also reflect a selection bias. For instance, families with children facing more complex or severe developmental issues may have been less likely to access clinical services during the pandemic due to health concerns, transportation barriers, or limited healthcare access.

In a large-scale US cohort study involving over 50,000 children aged 0–5 years, modest but statistically significant declines were observed in developmental screening scores during the pandemic—especially in communication, problem-solving, and personal-social domains—while motor domains remained relatively stable^17^. These findings mirror our results, which also point to domain-specific vulnerabilities, particularly in language and personal-social skills, suggesting that not all developmental areas are equally affected by environmental disruptions.

Another notable finding was the relative improvement in fine motor performance among children evaluated during the pandemic. This trend aligns with international evidence suggesting that increased time spent at home may have provided children with more opportunities for independent activities that enhance manual dexterity (11, 12 ). Activities such as tablet use, drawing, and other fine motor tasks may have replaced traditional forms of play during lockdowns—especially in households with access to educational resources and structured routines that facilitated such engagement. While increased screen time is often criticized for its potential negative effects on social interaction, emerging data indicate that touchscreen use and game-based motor tasks may have inadvertently supported fine motor development^11^. Moreover, some studies suggest that early and structured use of tablets correlates with improvements in advanced fine motor planning and visual-motor integration, which may further account for the observed preservation or enhancement of fine motor skills during this period^12^.

A longitudinal study from Portugal found that children assessed before and after the pandemic exhibited significant declines in global motor skills, yet showed improvement in fine motor abilities^18^. In contrast, our findings indicated no significant decline in gross motor development and a relative improvement in fine motor performance. These discrepancies may reflect cultural or contextual differences in how children’s physical activity opportunities were maintained during lockdowns—for example, through informal indoor play or continued movement within the home environment.

Several studies conducted during the pandemic have similarly reported that girls, particularly in early childhood, may be more adversely affected than boys in terms of mental health and developmental outcomes, although some have found no significant gender differences. Among adults, the findings are more mixed, with most research suggesting relatively equal levels of psychological impact across genders^19^.

In our sample, approximately two-thirds of the children who underwent developmental assessment were male, consistent with the well-established male predominance in referrals for neurodevelopmental concerns such as autism spectrum disorder (ASD) and attention-deficit/hyperactivity disorder (ADHD)^20^. Despite this higher referral rate among boys, our findings revealed that developmental delays during the pandemic period were more pronounced in girls, particularly in the language and personal-social domains, mirroring findings from Italian and Canadian studies suggesting that they may be especially sensitive to reductions in social engagement and emotional reciprocity during periods of isolation.

The notable increase in developmental delay rates among girls in our study underscores the importance of early identification, targeted interventions, and gender-sensitive risk management strategies during public health crises. This pattern may be partially explained by the “female protective effect,” which posits that females require a higher threshold of risk to display neurodevelopmental symptoms and, when affected, may present with more severe internalizing difficulties^20^.

Additionally, the breakdown of compensatory mechanisms such as camouflaging—more frequently observed in females—may have contributed to the heightened visibility of developmental concerns under pandemic conditions.

Finally, the contrasting trend of reduced fine motor delays, as opposed to increased delays in language and personal-social domains, may reflect differences in how environmental stressors influence specific developmental areas—potentially mediated by sex-specific neuroimmune responses^20^.

Our findings showing an increase in personal-social developmental delays during the pandemic period may reflect the sensitivity of this domain to environmental and psychosocial stressors. In line with this, a large-scale Turkish community study found that personal-social development was the least impaired domain under typical (non-pandemic) conditions, with only 4% of children showing delays in this area^21^. This contrast suggests that the increase we observed during the pandemic may be attributable to unique contextual disruptions, such as reduced peer interactions, limited outdoor play, and increased parental stress. These factors are likely to have disproportionately impacted children’s social-emotional development compared to other domains.Moreover, the relatively low rate of personal-social delay in the pre-pandemic reference sample strengthens the argument that the pandemic introduced new, significant challenges to this area of development.

Beyond behavioral observations, emerging studies suggest that COVID-19 may have direct neurobiological effects on child development. Although our dataset lacks infection status, prior research has identified white matter changes and inflammatory markers in children post-infection, with potential long-term implications (9, 10 ). This raises the possibility that some children in our sample may have experienced subtle cognitive or behavioral effects not fully detected by standard screening tools.

A prospective cohort study of neonates infected with the SARS-CoV-2 Delta variant reported significantly lower psychomotor development scores at 18–24 months compared to controls^22^. While our participants were not infected in the neonatal period, similar developmental concerns—particularly in language and personal-social domains—were observed, highlighting the broader risks posed by the pandemic environment.

In our study, the prevalence of global developmental delay as measured by the Denver II reached approximately 30% of the sample. This rate is considerably higher than those reported in large-scale community-based studies. For example, a Norwegian cohort study using the Ages and Stages Questionnaire reported suspected delays in only 5–7% of infants in the first year of life^23^. In the United States, national estimates suggest that approximately 16% of children present with some form of developmental disability, with specific delays such as language or motor difficulties affecting 5–10%^24^. By contrast, two national surveys from Egypt reported prevalence rates of around 6–7% among preschool-aged children^25^, while a study conducted in primary care centers in Turkey found a prevalence of 6.4%^26^.

The higher prevalence observed in our cohort most likely reflects the clinical nature of the sample, as children referred to a child and adolescent psychiatry clinic are enriched for developmental concerns. Importantly, there is limited literature on the prevalence of global developmental delay in clinical settings, representing a gap in current knowledge. Additionally, the psychometric limitations of the Denver II must be considered: while the instrument is highly sensitive in detecting potential delays, it has relatively low specificity, which may contribute to over-identification when compared to confirmatory diagnostic tools. Taken together, these factors likely explain the elevated prevalence observed in our study.

As a final point, while no significant differences were observed in gross motor domain between the pre-pandemic and pandemic periods, a reduction in fine motor delays was noted. Most notably, language-cognitive-social development emerged as the most adversely affected areas, with a threefold increase in the rate of isolated language delays during the pandemic. These findings underscore the heightened sensitivity of development to disruptions in social interaction, environmental stimulation, and intra-familial communication—factors significantly impacted by pandemic conditions.

## Conclusion

This study provides clear evidence that the COVID-19 pandemic has had differential effects on early childhood development. While language development appears to have been particularly vulnerable—especially in preschool-aged children—fine motor skills showed resilience, and in some cases, even improvement. These findings suggest that environmental disruptions such as social isolation, caregiver stress, and reduced peer interaction may selectively impact developmental domains, with lasting implications for language acquisition.

In contrast, the stability observed in gross motor and personal-social skills may reflect contextual buffers such as family support or continued opportunities for physical play. The disproportionate effect on language development underscores the importance of early screening and targeted interventions, especially for children born or raised during the pandemic.

As societies continue to recover from the effects of COVID-19, it is crucial to recognize and address the developmental scars left in its wake. Future public health strategies should consider not only infection control but also the holistic developmental needs of children, with a focus on restoring rich linguistic, social, and emotional environments during early life.

## Limitations

This study is limited by its single-center design and retrospective nature. As data were obtained from the only child psychiatry outpatient clinic in the region, the findings may not be generalizable to other populations and settings. No data were available regarding socioeconomic status, screen time, covid-19 infection history or caregiver stress levels, which could mediate developmental outcomes. Despite these limitations, the relatively stable population characteristics of the study region and the use of a standardized tool (Denver II) strengthen the internal validity of the findings. Furthermore, because we used a cumulative delay score rather than the standard categorical interpretation of the Denver II, the findings should be interpreted with caution.

In addition, the 2019 and 2021 cohorts consisted of independent samples rather than repeated assessments of the same children, which precludes longitudinal comparisons. Although no significant differences were found between groups in terms of age or sex, these factors may still have influenced developmental outcomes. However, given the restricted age range (0–6 years) and comparable group characteristics, additional statistical adjustment was not performed. Additionally, the Denver II is primarily a screening tool rather than a diagnostic instrument, and it has recognized psychometric limitations, including variable sensitivity and specificity across domains. These limitations may restrict the precision of developmental delay estimates in this study, and the findings should therefore be interpreted with appropriate caution.

## Data Availability

The datasets generated and analyzed during the current study are available from the corresponding author on reasonable request.
